# Dopaminergic manipulation modulates frequency-specific EEG connectivity patterns: evidence from a single dose drug challenge study

**DOI:** 10.3389/fnins.2026.1734025

**Published:** 2026-03-12

**Authors:** Alina J. Preuss, Renate de Bock, Amatya J. Mackintosh, Simon Schuster, Philipp Sterzer, Jonas Obleser, Stefan Borgwardt, Christina Andreou

**Affiliations:** 1 Translational Psychiatry Unit, Department of Psychiatry and Psychotherapy, University of Lübeck, Lübeck, Germany; 2 Center of Brain, Behavior, and Metabolism (CBBM), University of Lübeck, Lübeck, Germany; 3 Department of Psychiatry (UPK), University of Basel, Basel, Switzerland; 4 Department of Clinical Psychology and Epidemiology, Faculty of Psychology, University of Basel, Basel, Switzerland; 5 Department of Neurology, Asklepios Hospital Barmbek Hamburg, Hamburg, Germany; 6 Department of Psychology, University of Lübeck, Lübeck, Germany

**Keywords:** connectivity, dopamine, EEG, haloperidol, ICMS, L-dopa, resting-state

## Abstract

**Introduction:**

Dysregulation in resting-state connectivity in electroencephalogram (EEG) and dysregulation in the dopamine system have both been repeatedly observed in patients with psychotic disorders. It is unclear whether dopaminergic drugs, used to treat psychotic disorders, induce treatment-relevant connectivity changes in the brain. Investigating the effects of dopaminergic drugs in healthy participants could provide crucial insights into how dopaminergic modulation directly influences resting-state connectivity.

**Methods:**

In a randomized controlled crossover study including 58 healthy participants, we examined the effects of L-dopa (dopamine pre-cursor), haloperidol (dopamine D2 antagonist), and placebo on frequency-specific connectivity patterns (intrinsic coupling modes; ICMs). Source-space connectivity between brain regions was calculated for phase ICMs via multivariate interaction measure, and for envelope ICMs via orthogonalized power correlation.

**Results:**

Phase ICMs showed a significant linear increase (from L-dopa to placebo to haloperidol) in the delta frequency, and a significant linear decrease in the low-beta frequency. Gamma phase ICMs were significantly reduced in both drug conditions compared to placebo. Orthogonalized power correlation showed a significant linear decrease (from L-dopa to placebo to haloperidol), in the alpha and low-beta frequency.

**Discussion:**

Our findings in the gamma band support the long-standing hypothesis that dopaminergic action on large-scale neural networks follows an inverted U-shaped curve. The linear pattern of our findings in the delta, alpha, and low-beta frequency bands reflect possible relevant effects for psychotic symptoms. However, our findings are difficult to relate to existing findings in patients. Our results highlight the need for further prospective studies in patients to better understand the relationship between dopaminergic effects and ICMs.

## 1 Introduction

The discovery of antipsychotics in the 1950s proved to be important in treating patients with psychotic disorders, significantly reducing the severity of psychotic symptoms such as hallucinations. Most antipsychotic drugs have a shared mode of action: the antagonism of the dopamine D2 receptor. This is in support of the dopamine hypothesis, which, in a simplified form, postulates that higher levels of striatal dopamine are associated with psychotic symptoms such as hallucinations and delusions (Howes and Kapur, 2009). While antipsychotics are often effective in treating positive symptoms, they are less effective for negative and cognitive symptoms. In fact, more recent studies suggest that antipsychotics may worsen cognitive symptoms (Husa et al., 2014; Allott et al., 2024). Consequently, it is crucial to enhance our understanding of the mechanisms through which antipsychotics exert their effects in the brain.

In this context, the dysconnectivity hypothesis gives a perspective on the pathophysiology of psychotic disorders (Friston et al., 2016). This hypothesis includes both increases and decreases in functional connectivity between brain regions. Dysconnectivity is commonly observed in both fMRI and EEG studies that include patients with psychotic disorders (Northoff and Duncan, 2016; Dong et al., 2018; Cai et al., 2022). In contrast to fMRI’s high spatial resolution, EEG has a high temporal resolution and can capture oscillations of neural interactions at the timescales at which they appear, being able to capture dynamics that occur in milliseconds. Therefore, EEG is a powerful tool for investigating the temporal aspects of brain activity, enabling a detailed analysis of the brain’s network components.

Although the dopamine hypothesis and the dysconnectivity hypothesis are both well-researched, only several studies have simultaneously investigated these two together. In a recent review, Mackintosh et al. (2021) summarized findings on the relation between resting-state connectivity in psychotic disorders and antipsychotic medication. The authors reported conflicting findings across studies, largely explained by a wide heterogeneity in methodological approaches. The authors identified the lack of longitudinal studies directly investigating the effects of dopaminergic drugs on EEG resting-state connectivity as a significant gap in the literature. Placebo-controlled studies in healthy participants can provide systematic insights into the effects of dopaminergic drugs on resting-state connectivity.

Resting-state EEG measures ongoing brain activity, which can be described in terms of intrinsic coupling modes (ICMs). Engel et al. (2013) proposed two types of ICMs in ongoing brain activity to describe connectivity: phase ICMs, reflecting phase coupling (e.g., coherence, imaginary coherence), and envelope ICMs, measuring signal envelope correlation (amplitude or power correlation) as coupling measure. The above distinction does not only have theoretical value; the two types of coupling mode have quite different attributes [reviewed in Engel et al. (2013)]. Phase connectivity measures are affected by state factors such as stimulus context or cognitive setting, and are strongly susceptible to global state changes such as sleep (Supp et al., 2011). Envelope ICMs have been linked to fMRI BOLD fluctuations and correlate more clearly with structural connectivity (Wang et al., 2013; Messé et al., 2023). It has been suggested that the two types of connectivity serve distinct but complementary purposes, with envelope ICMs exerting a regulatory role by determining the availability of neuronal populations, and phase ICMs supporting selective communication between neuronal populations. In this framework, multiple phase ICMs may be nested within a single envelope ICM-defined network (Engel et al., 2013). Together, these coupling modes provide complementary insights: phase-coupling reveals the fine-grained timing of neural communication, while amplitude-coupling uncovers the broader architecture and long-range connectivity of brain networks. This dual perspective is essential for a comprehensive understanding of how neuronal interactions support complex brain functions (Siems and Siegel, 2020).

To the best of our knowledge, only one previous study assessed the effects of dopaminergic drugs on EEG connectivity in healthy participants. Albrecht et al. (2016) showed that after dexamphetamine (an indirect dopamine agonist) administration, connectivity measured with phase ICMs was increased in the parietal, occipital-temporal, and occipital regions in the alpha and theta frequency band, while orthogonalized power correlation, a measure of envelope ICMs, remained unaffected. However, a full investigation of dopaminergic effects on connectivity should include increasing dopaminergic transmission as well as decreasing dopaminergic transmission.

Converging evidence suggests that the relationship between cortical activity and dopaminergic transmission follows an inverted U-shape, such that both hypo- and hyperdopaminergic extremes can lead to impaired performance, e.g., on cognitive functions (Williams and Castner, 2006; Cools and D’Esposito, 2011). Using both dopamine-enhancing as well as dopamine-reducing manipulations helps to discern effects that may follow a linear pattern, potentially relevant for the pathophysiology and treatment of psychotic symptoms, from drug effects on networks supporting cognitive functions, which might exhibit a quadratic effect.

Here, we investigated the effects of a single-dose of L-Dopa (a dopamine pre-cursor) and haloperidol (a dopamine D2 antagonist) on phase and envelope ICMs in healthy participants. We used a randomized, placebo-controlled, double-blind, three-way cross-over design. We hypothesized that dopaminergic modulation would alter resting-state connectivity in both linear and non-linear patterns. Based on the aforementioned findings by Albrecht et al. (2016) as well as previous studies by our own group in patients with psychotic disorders and high risk subjects (Andreou et al., 2015a,b), we expected to observe significant effects of the dopaminergic manipulation mainly in the theta, alpha and gamma frequency bands.

## 2 Materials and methods

### 2.1 Participants

We included healthy participants between 18 and 40 years (*N* = 61). The participants were recruited via the student website of the University of Basel, advertisements on the university’s online marketplace, and by word-of-mouth. Exclusion criteria comprised a past or current psychiatric or neurological disorder, including substance abuse [assessed with the Mini International Neuropsychiatric Interview (Sheehan et al., 1998)], a history of schizophrenia or a bipolar disorder in a first-degree relative, a history of cranio-cerebral trauma, arterial hypertension, cardiological or serious medical conditions, pregnancy or breastfeeding and current treatment with any (psychotropic or other) drug (including hormonal contraceptives).

Written informed consent was obtained and participants were reimbursed (money and/or course credit) for their participation. The study was approved by the local ethics committee (Ethikkommission Nordwest- und Zentralschweiz, registration number 2016-01734).

### 2.2 Study design and procedure

The study was carried out in a randomized, double-blind, three-armed crossover design. In three successive visits, separated by a minimum of 7 days to guarantee total substance washout, participants received capsules with either 100 mg of L-dopa and 25 mg benzaseride (Madopar), 2 mg haloperidol (Haldol), or placebo. The dose of L-dopa we used in this study is identical to that used in previous studies in healthy participants (Angwin et al., 2004; Copland et al., 2009; Kelly et al., 2009; Andreou et al., 2015c). The dose of haloperidol corresponds to a D2-receptor occupancy of approximately 70%, which is deemed sufficient for a clinical response while minimizing adverse effects (Kapur et al., 1997, 2000).

All substances were administered in visually identical capsules prepared by the Pharmacy of the University Hospital Basel, and a medical doctor supervised each session. To ensure that resting-state EEG recordings consistently took place at peak drug concentration, a double-dummy design was used (Supplementary Table 1). EEG recordings began approximately 2.5 h after the start of the experimental session.

The pharmacokinetic variability of L-dopa can be reduced by standardizing intake with respect to mealtimes and protein consumption (Contin and Martinelli, 2010). When eating protein, dietary amino acids compete with L-dopa for the carrier system across the intestinal mucosa, which affects L-dopa absorption. To minimize this source of variability across sessions and participants, participants were instructed to adhere to a low-protein diet on the day of the session until 1 h before the session, and to refrain from any food or caffeine intake until the end of the session. At the end of each session, participants were asked to guess which substance they had received.

### 2.3 EEG data collection

The EEG recordings were carried out at the Neuroscience Userlab of the Faculty for Psychology at Basel University. We used 64 electrodes positioned according to the international 10-10 system, using the BioSemi ActiveTwo system. The sampling rate was set at 1024 Hz, and electrode impedance was maintained below 25 kΩ. To monitor eye movements, four electrooculogram (EOG) channels were placed–two horizontally at the temples and two vertically above and below the left eye. Participants sat in a quiet, light-shielded room, where they underwent a 10-min session of an eyes-closed resting-state recording. To reduce the risk of drowsiness, an audio cue through headphones instructed participants to open their eyes for 10 s every 2 min.

### 2.4 EEG preprocessing and analysis

Offline preprocessing was performed with BrainVision Analyzer (Version 2.2.2, Brain Products GmbH, Gilching, Germany). We applied a 50 Hz notch filter (24 dB/octave, zero-phase lag) and a 0.5–70 Hz IIR bandpass filter (12 dB/octave, zero-phase lag). After manually eliminating segments with large artifacts, channels with poor quality across the entire recording were interpolated (max 10%). Extended infomax ICA was used to remove ocular artifacts. The data were segmented into 2-s epochs, and epochs with remaining artifacts were removed using semi-automatic artifact rejection (maximal gradient: 50 μV/ms, Max-Min difference: 200 μV, low activity: 0.5 μV). A minimum of 30 artifact-free epochs was required for inclusion in the analyses. This criterion was met for all recording sessions. The mean number of artifact-free epochs was 226.18 ± 38.58 in the placebo condition, 225.91 ± 40.50 after L-DOPA, and 219.33 ± 41.16 after haloperidol. We downsampled the data to 256 Hz and re-referenced it to the average reference.

Spectral estimates were obtained within consecutive time windows with a 75% overlap. The window length ranged from 0.125 to 0.5 s, depending on the wavelet’s center frequency, to achieve a frequency resolution of 2 Hz for the delta and theta bands, 4 Hz for the alpha to high beta bands, and 8 Hz for the gamma band. The center frequencies of the wavelet were set at 3, 6, 10, 16, 25 and 40 Hz for the delta, theta, alpha, low-beta, high-beta, and gamma frequency range, respectively.

Scalp-level EEG data were transformed to source space using eLORETA (Pascual-Marqui et al., 2011), which estimates the underlying cortical activity by solving the inverse problem. The head-surface EEG was recomputed into 80 source model time-series. These correspond to the centers of all cortical and hippocampal regions of the automated anatomical labeling (AAL) atlas (Tzourio-Mazoyer et al., 2002), with 40 sources in each hemisphere. Connectivity measures are therefore calculated on (80^2^−80)/2 = 3160 pairs of sources. Coordinates for the 80 cortical points used for connectivity analyses were defined based on the standard METH toolbox template, using the grid points that were closest to the local maxima of respective AAL regions (see Supplementary Table 2).

As described before, there are two types of intrinsic coupling modes (ICMs) in ongoing brain activity that can be used to describe connectivity: envelope and phase ICMs (Engel et al., 2013). For each participant and substance (L-dopa, haloperidol, and placebo), we created connectivity matrices based on the phase and envelope intrinsic coupling modes. To investigate phase ICMs, we used the multivariate interaction measure (MIM) (Ewald et al., 2012). For each grid point, MIM considers that data at the source level are 3-dimensional, corresponding to 3 dipole orientations and that activity at a given point also reflects neural activation of the points in the vicinity. For envelope ICMs, we used orthogonalized power correlation (ORT) (Hipp et al., 2012). Analyses were performed in Matlab (Mathworks, R2021b) using the MEG and EGG Toolbox of Hamburg (METH).^1^ METH is a collection of MATLAB functions.

As the analysis of EEG resting-state data is prone to the multiple-comparisons problem due to the immense quantity of calculations, we used Network-Based Statistics (NBS) (Zalesky et al., 2010), a type of cluster-based permutation testing to identify networks of aberrant connectivity. We set the significance level to 0.05 and the number of permutations to 5000 for the analysis of all frequency bands and calibrated the primary t-threshold in the range of 2.3–3.8 in intervals of 0.1. We used pre-defined polynomial contrasts across the three conditions (L-dopa, placebo, haloperidol), testing for linear and quadratic trends.

In cases where participants were missing one or two EEG datasets, all available data were processed using the standard preprocessing pipeline up to the network-based statistic (NBS) stage. Because of the repeated-measures (crossover) design, the software does not allow for incomplete datasets in the design matrix and incomplete datasets were not included in the network analysis. A detailed description of excluded datasets is provided in the section “3 Results.”

It is important to note that EEG signals represent a combination of both genuine oscillatory (i.e., periodic narrowband) activity and aperiodic activity, and it has been suggested that the two components have different physiological functions (Donoghue et al., 2020). EEG-analyses based on canonical frequency bands may confound the effects of experimental factors on periodic and aperiodic components, potentially affecting the interpretation of findings; for example, a shift in the center frequency of oscillations, or changes in the aperiodic component properties, may lead to spurious differences in oscillatory power within a canonically defined frequency band (Donoghue et al., 2020). To exclude that such effects may have affected our findings, we computed spectral density for the frequency range at a resolution of 1 Hz at the source level based on the 80 aforementioned eLORETA source model time-series, and averaged these over the nodes of networks, for which significant connectivity differences were observed. Subsequently, we used the FOOOF (fitting oscillations & one over f) toolbox v1.1.1 (Donoghue et al., 2020) to compare parameters of network power spectra across substances. For details, please refer to the Supplementary materials.

### 2.5 Vigilance

Changes in arousal during resting-state measurements are possible by natural variation, but also because of dopaminergic manipulation. We controlled for possible changes in arousal across conditions by comparing different stages of vigilance. We used VIGALL 2.1^2^ as an add-in for BrainVision Analyzer. The different stages are state A (combined A1–A3; wakefulness), state B (B1–B2/3; drowsiness), and state C (C; sleep). To quantify the vigilance levels, we calculated the relative number of 1-s segments that fell within each stage for each participant and condition. Specifically, we counted the number of segments that fell into each stage and divided by the total number of segments in the task, expressed as a percentage.

### 2.6 Statistics

To investigate substance effects in healthy participants, we included the substance (L-dopa, haloperidol, placebo) as a repeated effect and defined linear and quadratic contrasts in the connectivity analysis. To examine the distribution of the connectivity and to visualize the effects, we extracted mean connectivity values from the significant networks of the NBS analysis. *Post hoc* paired *t*-tests with Holm-adjusted *p*-values were then used to assess substance effects on these mean connectivity values. All statistical analyses were conducted in R (version 4.4.3)^3^ using R-Studio (version 2024.12.1.563)^4^.

To exclude power differences as a cause for differences in connectivity, we calculated mean eLORETA scalp oscillatory power across all 80 sources, and conducted a 3 (substance) × 6 (frequency-band) repeated-measures ANOVA.

## 3 Results

### 3.1 Participants

Of the sixty-one participants who originally provided informed consent, *n* = 2 did not meet the inclusion criteria, *n* = 1 did not adhere to the study protocol and *n* = 4 withdrew consent after the first, second or third recording session. This resulted in fifty-four complete datasets that were included in the analysis (Supplementary Figure 1). See Table 1 for a detailed description of participant characteristics. Blinding to the substances was preserved as we found no significant relationship between the ingested and guessed substances [X^2^(2) = 3.30, *p* = 0.192].

**TABLE 1:** Participant characteristics.

Variable	*N* = 54
**Sex, *n* (%)**	
Female	25 (46%)
Male	29 (54%)
**Age, mean (SD)**	25.2 (4.6)
**Handedness, *n* (%)**	
Left	7 (13%)
Right	47 (87%)
**School years, mean (SD)**	12.32 (2.14)
**Further education, *n* (%)**	
No further degree	22 (42%)
Apprenticeship	4 (7.5%)
University of applied sciences	2 (3.8%)
University	25 (47%)
Unknown	1
**Civil status, *n* (%)**	
Single	40 (74%)
Relationship	11 (20%)
Married	3 (5.6%)
**Employment, *n* (%)**	33 (61%)
**Alcohol use, *n* (%)**	39 (72%)
**Nicotine use, *n* (%)**	7 (13%)
Cannabis use, *n* (%)*	1 (1.9%)

*One participant reported using *Cannabis* approximately once a month; the urine drug screening at the time of each visit was negative.

### 3.2 Power

The main effect of substance on scalp oscillatory power was not significant [*F*(2,901) = 0.228, *p* = 0.796], and neither was the substance × frequency-band interaction [*F*(10,901) = 0.163, *p* = 0.998]; Supplementary Figure 2).

### 3.3 Phase ICMs (MIM)

#### 3.3.1 Delta

**FIGURE 1:**
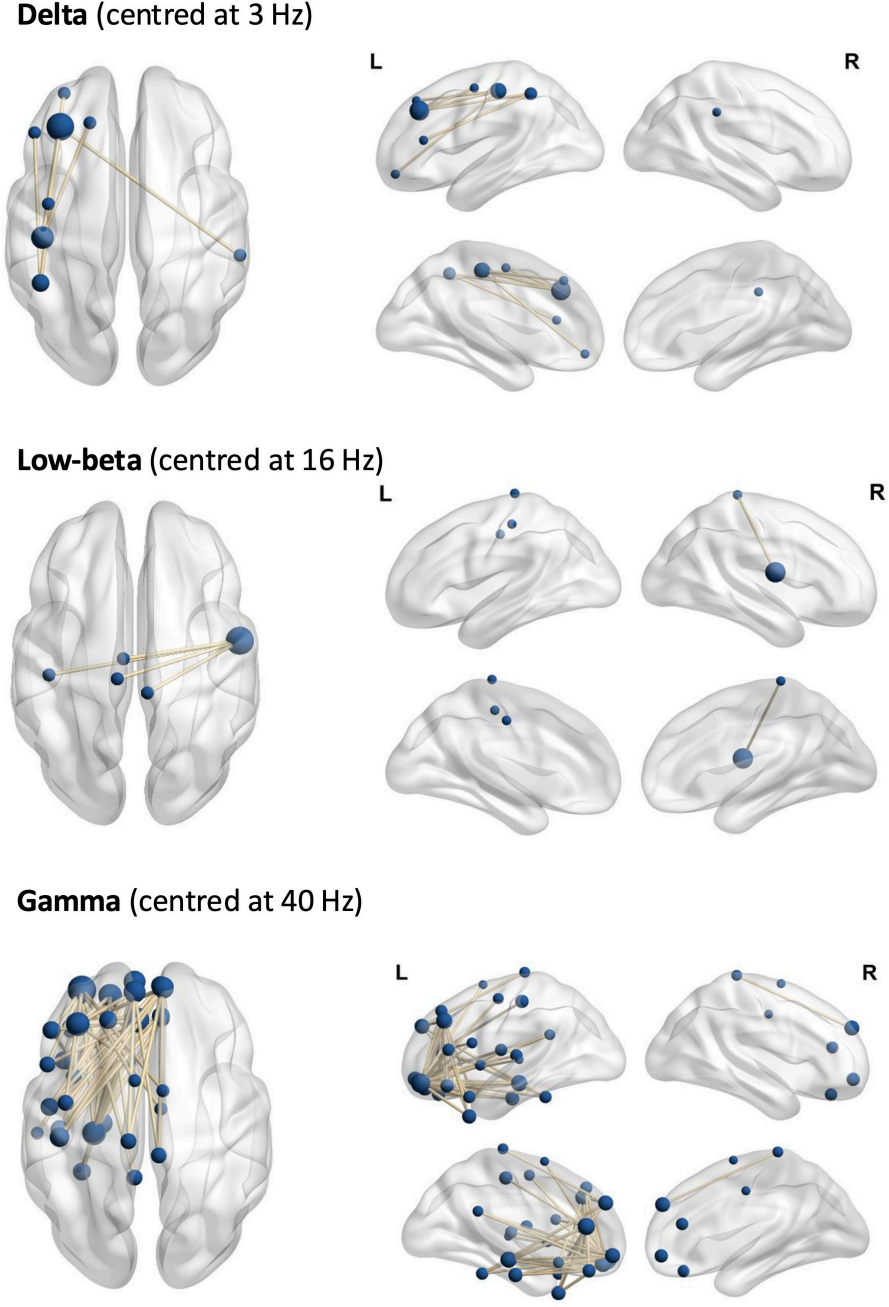
The networks of significant phase coherence (MIM). Top: delta, middle: low-beta, bottom: gamma.

**FIGURE 2:**
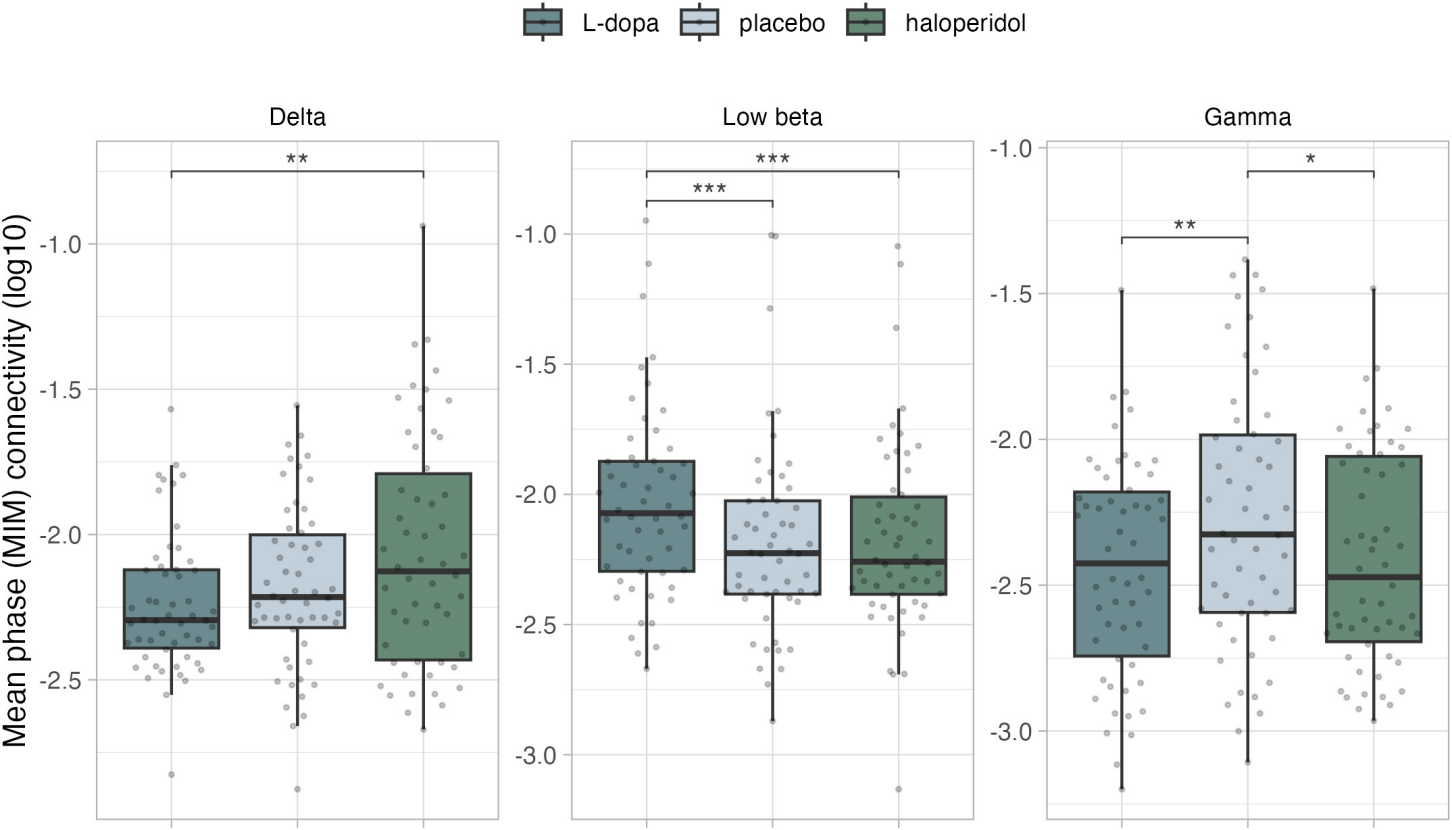
Mean phase coherence (MIM) of the significant networks per frequency band. *p < 0.05, **p < 0.01, ***p < 0.001 (Holm adjusted). Data were log-transformed for illustration purposes, but not for analyses.

In the delta frequency, NBS revealed a significant network (threshold *t* = 3, p_adj = 0.039), encompassing 7 edges and 8 nodes, mainly in left fronto-parietal regions (see Figure 1 and Supplementary Table 2). We observed a significant linear trend in mean connectivity, with connectivity increasing from L-dopa to placebo to haloperidol. This network exhibited higher mean MIM in the haloperidol condition, compared to placebo [t(53) = −2.72, p_adj = 0.018] and L-dopa [t(53) = −3.04, p_adj = 0.012] (Figure 2).

#### 3.3.2 Low-beta

In the low-beta frequency range, we found a significant network (threshold *t* = 3.4, p_adj = 0.034), encompassing 4 edges and 5 nodes around the left and right paracentral lobule (Figure 1 and Supplementary Table 2). More specifically, a significant linear effect emerged, with connectivity decreasing from L-dopa to placebo to haloperidol. This network exhibited higher mean MIM in the L-dopa condition, compared to placebo [t(53) = 3.34, p_adj = 0.004] and haloperidol [t(53) = 4.29, p_adj < 0.001] (Figure 2).

#### 3.3.3 Gamma

In the gamma frequency range, we found a significant network (threshold *t* = 2.9, p_adj = 0.009), encompassing 68 edges and 33 nodes. The network encloses predominantly left frontotemporal cortex, including the left postcentral lobule and the left supplementary motor area node (Figure 1 and Supplementary Table 2). A significant quadratic effect was found, with significant lower mean MIM in both substance conditions compared to placebo [vs. L-dopa t(53) = −3.49, p_adj = 0.003; vs. haloperidol t(53) = 3.39, p_adj = 0.003] (Figure 2).

#### 3.3.4 Other frequency bands

There were no significant networks in the theta, alpha, or high-beta frequencies (all *p* > 0.05). For visualization purposes, we additionally show condition-specific MIM networks using one-sample NBS *t*-tests within each condition (1,000 permutations; Supplementary Figure 3). These maps are descriptive and were not used for between-condition statistical tests.

### 3.4 Envelope ICMs (ORT)

#### 3.4.1 Alpha

**FIGURE 3:**
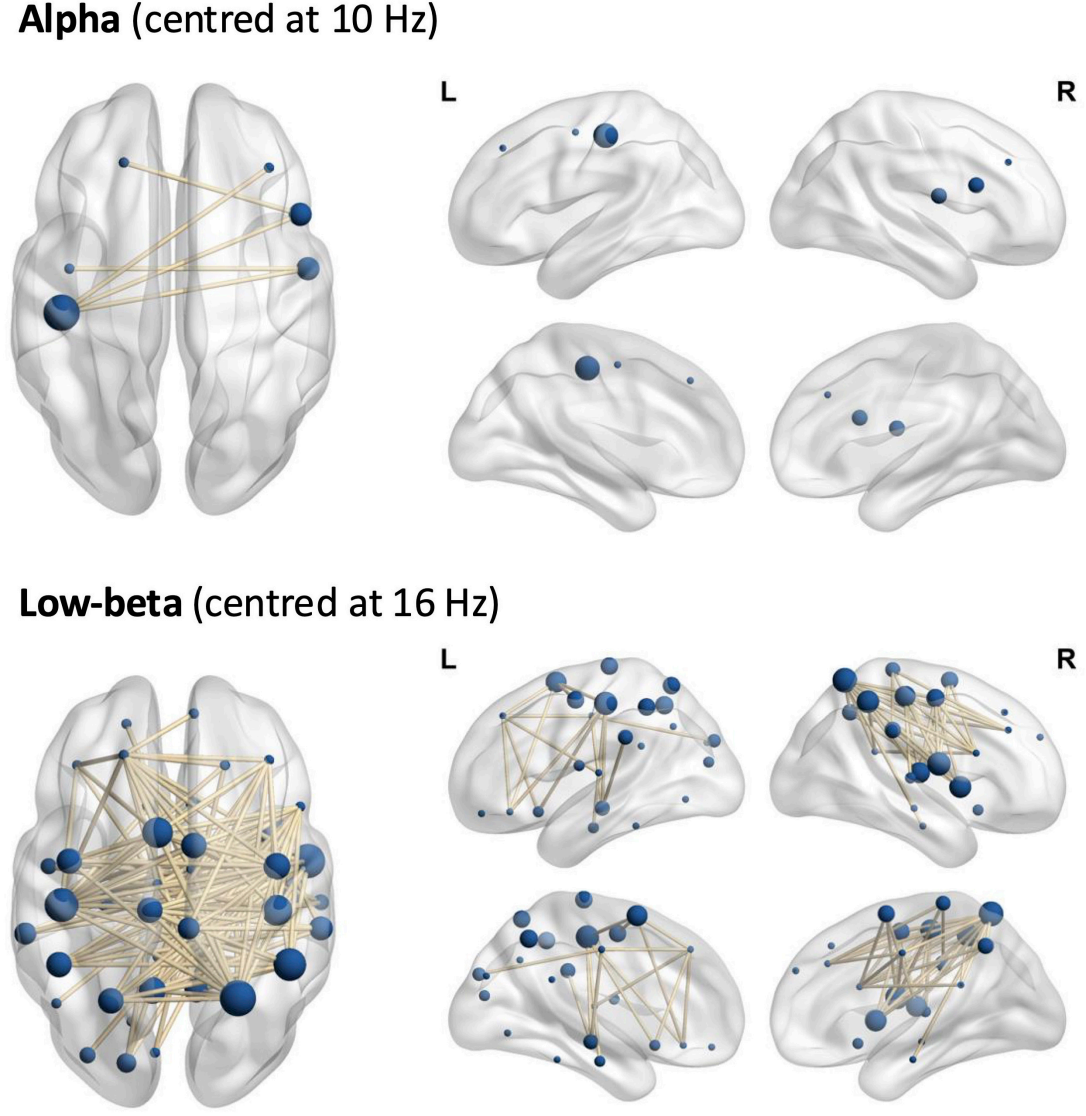
The networks of significant envelope coherence (ORT). Top: alpha, bottom: low-beta.

**FIGURE 4:**
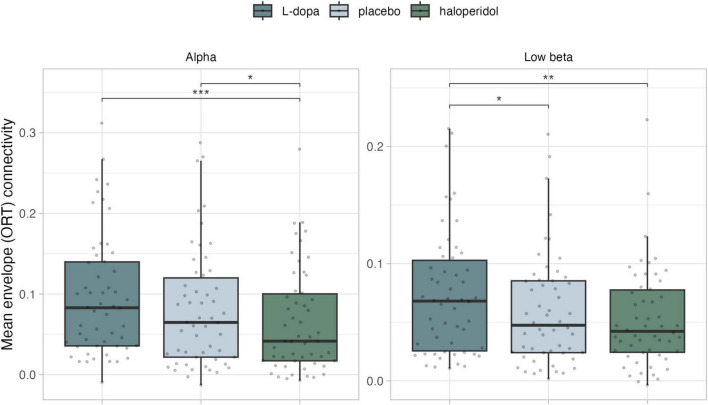
Mean phase coherence (ORT) of the significant networks per frequency band. *p < 0.05, **p < 0.01, ***p < 0.001 (Holm adjusted).

In the alpha frequency range, we found a significant network (threshold *t* = 3.4, p_adj = 0.036), encompassing 5 edges and 6 nodes in the frontal and parietal lobes (Figure 3 and Supplementary Table 3). Again, a significant linear contrast was observed, with connectivity decreasing from L-dopa to placebo to haloperidol. This network exhibited lower mean ORT in the haloperidol condition, compared to placebo [t(53) = 2.66, p_adj = 0.020] and L-dopa [t(53) = 4.03, p_adj < 0.001] (Figure 4).

#### 3.4.2 Low-beta

In the low-beta frequency range, we found a significant network (threshold *t* = 2.4, p_adj = 0.049), encompassing 145 edges and 45 nodes in the prefrontal cortex, motor cortex, somatosensory cortex, cingulate cortex, insular cortex, temporal cortex, occipital cortex, and limbic structures (Figure 3 and Supplementary Table 3). More specifically, a significant linear contrast emerged, with connectivity decreasing from L-dopa to placebo to haloperidol. This network exhibited higher mean ORT in the L-dopa condition, compared to placebo [t(53) = 2.66, p_adj = 0.020] and haloperidol [t(53) = 3.22, p_adj = 0.006)] (Figure 4).

#### 3.4.3 Other frequency bands

There were no significant networks in the delta, theta, high-beta, or gamma frequencies (all *p* > 0.05). For visualization purposes, we additionally show condition-specific ORT networks using one-sample NBS *t*-tests within each condition (1,000 permutations; Supplementary Figure 4). These maps are descriptive and were not used for between-condition statistical tests.

### 3.5 FOOOF

Detailed FOOOF analysis results are presented in the Supplementary materials. Robust oscillatory power peaks were observed in the theta, alpha and low-beta range in all networks, but not in the gamma and delta range. There were no differences between substances in either the distribution of frequency peaks or aperiodic component parameters.

### 3.6 Vigilance

Across all participants and substances, state A and state B were about equally prevalent (49.73% and 50.27%, respectively). No significant interaction (substance × state) [*F*(2,318) = 0.09, *p* = 0.912] or main effect of substance on vigilance [*F*(2,318) < 0.001, *p* > 0.999] was observed.

## 4 Discussion

The aim of this study was to investigate the effects of dopaminergic manipulation on EEG resting-state connectivity using intrinsic coupling modes (ICMs) in healthy participants. For phase ICMs, we found significant linear effects in the delta band (increasing from L-dopa to placebo to haloperidol) and in the low-beta band (decreasing from L-dopa to placebo to haloperidol), and quadratic effects in the gamma band (decreased in both drug conditions). For envelope ICMs, we found significant linear effects in the alpha and low-beta band (decreasing from L-dopa to placebo to haloperidol).

### 4.1 Phase ICMs

Contrary to our hypothesis, we did not find changes in phase coherence in the theta band following dopaminergic manipulation. We expected to find an increase in theta band in the L-dopa condition based on previous findings. Albrecht et al. (2016) reported an increase in theta band phase coherence following the administration of the indirect dopaminergic agonist dexamphetamine. However, dexamphetamine and L-dopa have different mechanisms of action and likely affect phase coherence differently. L-dopa serves as a precursor to dopamine, while dexamphetamine more directly increases dopamine levels by promoting its release and inhibiting reuptake. In addition, dexamphetamine also increases glutamate (White et al., 2018), and effects could potentially be similar to the NMDA receptor antagonist ketamine, which is known to increase glutamate levels (Zhang et al., 2021). Indeed, ketamine has shown to increase theta band phase coherence in healthy participants (Curic et al., 2021). Some studies in patients with psychotic disorders show increased phase coherence in the theta band (Andreou et al., 2015a; Di Lorenzo et al., 2015; Umesh et al., 2018; Krukow et al., 2020). However, Andreou et al. (2015a) showed that this change was partially mediated by verbal memory deficits, and that high-risk patients showed intermediate patterns between those of patients and HC, suggesting a trait nature of theta-band coherence changes. Thus, changes in theta-band coherence might be the result of longer-term changes in dopaminergic activity or related to other neurotransmitter systems.

In the gamma frequency band, we observed significant quadratic effects, showing a decrease in gamma band phase coherence after administration of both L-dopa and haloperidol, compared to placebo. This finding aligns with the long-standing hypothesis that dopaminergic activity follows an inverted U-shaped pattern (Williams and Castner, 2006; Cools and D’Esposito, 2011), which are particularly evident in the gamma-band (Kömek et al., 2012). As mentioned before, phase coherence is closely linked to anatomically structured networks that can mirror patterns seen in fMRI studies. In this regard, a resting-state fMRI study with similar manipulations (L-dopa and haloperidol in healthy participants) reported an inverted U-shaped pattern in a resting-state network (Cole et al., 2013).

In research on patients with schizophrenia, findings in gamma band connectivity are inconsistent, largely attributable to significant methodological variations across study designs and populations (Maran et al., 2016; Mackintosh et al., 2021). One form of heterogeneity in study populations is the variability of cognitive function. For example in patients with first-episode psychosis, there is considerable heterogeneity in cognitive function (Lee et al., 2024). Cognitive function and processes are known to be impaired in psychotic disorders, and cognition is frequently associated with gamma band oscillations (Jensen et al., 2007; Tanaka-Koshiyama et al., 2020). Importantly, cognitive impairments in psychotic disorders are often not adequately addressed by antipsychotic treatments and may even be exacerbated by potent dopamine antagonism in extrastriatal areas such as the frontal cortex (Allott et al., 2024). Our findings are in line with these observations and provide a potential mechanism through which antipsychotics contribute to cognitive impairments. However, because our data were acquired at rest, we cannot directly link the observed connectivity changes to cognitive performance.

In the low-beta frequency band, we observed a significant linear decrease in phase coherence from L-dopa to placebo and haloperidol, whereas delta-band phase coherence showed the opposite pattern, i.e., a significant linear increase from L-dopa to placebo to haloperidol. Evidence from pharmaco-EEG source-level connectivity studies in healthy participants remains scarce; notably, the only closely comparable study using an indirect dopamine agonist (dexamphetamine) did not report changes in beta or delta connectivity (Albrecht et al., 2016).

We initially hypothesized that linear drug effects would be relevant for the pathophysiology and treatment of psychotic symptoms. However, findings in the existing literature that compare patients with psychotic disorders to healthy controls are highly inconsistent and studies observe connectivity changes in both directions or no differences in connectivity (Mackintosh et al., 2021). This heterogeneity likely reflects not only differences in connectivity metrics, but also methodological variability in EEG acquisition and preprocessing. Additional sources of variability include sample size, antipsychotic exposure (type and duration), and illness duration (Mackintosh et al., 2021). Moreover, the acute pharmacological effects observed here in healthy participants are not directly comparable to the dopaminergic alterations present in psychotic disorders. Nevertheless, the present results show that L-dopa and haloperidol can systematically shift phase coupling in specific frequency bands. A key next step will be longitudinal studies examining whether beta- and delta-band coupling changes are sensitive to antipsychotic treatment course and whether they covary with clinical and functional measures.

Although direct pharmaco resting-state EEG connectivity comparisons are limited, beta and delta frequency bands have clear functional associations in the EEG literature. Beta activity is commonly linked to sensorimotor processing (Barone and Rossiter, 2021; Böttcher et al., 2023), and the sensorimotor resting-state network is related to dopaminergic signaling (Conio et al., 2020). Delta activity is often discussed in relation to slow, large-scale fluctuations and state regulation. Task-based studies also show that delta is associated with predictive timing, and beta with the nature of upcoming stimuli (Engel et al., 2013). Importantly, these associations are primarily based on power measures. Connectivity is conceptually distinct, and power effects do not straightforwardly translate to changes in phase coupling. Therefore, rather than claiming a direct correspondence with prior power findings, our results add complementary evidence that dopaminergic manipulation can systematically modulate inter-regional phase coupling in beta and delta ranges. A next step will be longitudinal studies in patient groups to test whether coupling abnormalities in these bands are present and whether they change with antipsychotic exposure over time.

### 4.2 Envelope ICMs

In the alpha frequency band, we found a significant linear effect: orthogonalized power envelope connectivity decreased from L-dopa to placebo to haloperidol. The decrease in connectivity was most pronounced in the haloperidol condition compared to both L-dopa and placebo. A previous dopamine manipulation study in healthy participants did not observe changes in alpha band orthogonalized power coupling (Albrecht et al., 2016). However, that study used the dopamine agonist dexamphetamine which, as detailed above, has a different mode of action than L-dopa. In the low-beta frequency band, a similar linear pattern in the opposite direction was observed. Previous studies investigating envelope ICMs in patients with schizophrenia were carried out using fMRI (Engel et al., 2013), which makes it difficult to compare previous findings to the current work.

Alpha and low-beta frequency activity have been described as being strongest in deep layers of the cortex (van Kerkoerle et al., 2014; Michalareas et al., 2016; Scheeringa and Fries, 2019). Recently, a study in mice showed that antipsychotic drugs, including haloperidol, induce selective decorrelation of activity specifically in deep cortical layers (layer 5) (Heindorf and Keller, 2024). This finding may explain why a reduction along the dopamine gradient results in an observed linear decrease in connectivity within these frequency ranges in the present study.

### 4.3 Relationship between phase and envelope ICMs

Phase- and envelope-based ICMs are conceptually related yet provide non-redundant descriptions of large-scale coupling in ongoing brain activity. Phase-coupling involves the consistent alignment of the phase of neuronal oscillations, enabling frequency-specific interactions that support cognitive processes through precise temporal synchronization across brain regions (Siems and Siegel, 2020). In contrast, amplitude-coupling refers to the temporal co-modulation of the amplitude or power of neuronal oscillations, forming stable, large-scale networks by coordinating the strength of neural activity (Siems and Siegel, 2020). The neuronal mechanisms underlying these modes differ: phase-coupling emphasizes the timing and synchronization of neural signals, while amplitude-coupling reflects the organization and hierarchical coordination of network activity. Importantly, Siems and Siegel (2020) showed that the similarities and differences of the two coupling patterns depend on the frequency band. In the context of the present study, this implies that both measures are not necessarily affected in the same way by dopaminergic manipulation. Consistent with this, most of our effects were specific to one measure.

### 4.4 Strengths and limitations

There are several factors that contribute to individual differences in dopamine response. For example, genetic variation (Gluskin and Mickey, 2016), and as a result, personality traits (Suhara et al., 2001), value-based decision-making (Dang et al., 2018; Banuelos et al., 2024), and addiction risk (Zald, 2023) are all factors that can influence dopamine functioning. However, a key strength of the present study is its randomized, double-blind, cross-over, placebo-controlled design, which minimizes the impact of inter-individual differences in dopamine functioning by enabling within-subject comparisons. To study the effects of dopaminergic drugs, we selected drugs expected to enhance dopamine signaling (L-dopa) as well as drugs expected to reduce dopaminergic signaling (haloperidol). However, these drugs act via distinct mechanisms and should not be interpreted as having opposite effects. L-dopa is not a specific dopaminergic receptor agonist, but a dopamine precursor. L-dopa has a complex pharmacology: dopamine formed from exogenous L-dopa can be produced and released by serotonergic neurons, which may result in a more widespread dopaminergic signal than endogenous transmission, and actions beyond conversion to dopamine cannot be excluded (e.g., via GPR143) (De Deurwaerdère et al., 2017). Therefore, the effects of L-dopa are widespread. Haloperidol is a D2 receptor antagonist, and the D2 receptor is widely distributed throughout the brain. Thus, D2 receptor blockade by haloperidol affects a wide range of striatal, limbic and cortical regions (Xiberas et al., 2001; Wächtler et al., 2020). Further research could include a broader range of antipsychotic medications. Studying healthy participants is an additional strength, as it allows dopaminergic effects on brain connectivity to be examined without confounding influences of illness, medication history, or disease-related neural alterations. However, it is important to note that single-dose drug challenge studies differ from studies conducted in patients receiving stable treatment. Given the inconsistent findings in studies involving patients with psychotic disorders, longitudinal studies are needed to better elucidate the dopaminergic effects on intrinsic connectivity and their relation to psychotic disorders.

Several methodological constraints inherent to EEG should also be considered. For example, 50-Hz notch filters have been criticized for introducing artifacts and spectral distortions in the gamma band (Leske and Dalal, 2019). These effects should mainly affect time-locked measures such as ERPs and Granger causality (Widmann et al., 2015; Leske and Dalal, 2019). Moreover, the design of our notch filter is expected to create minimal distortion in the frequency range of interest in this study (36–44 Hz). Nevertheless, our results regarding gamma range connectivity should be interpreted with caution. Although our approach of using canonical frequency bands is common in EEG research, it also has limitations. Using FOOOF, we confirmed the presence of discernible oscillatory peaks in the theta, alpha, and beta ranges, whereas no clear peaks were identified in the delta or gamma ranges. Importantly, the absence of a peak above the aperiodic component does not necessarily rule out the presence of oscillatory activity (Donoghue et al., 2020). Moreover, the aperiodic components did not differ between conditions, suggesting that our results are robust and unlikely to be driven by condition-related differences in the aperiodic signal. Finally, the use of connectivity measures that removed zero-lag connectivity to avoid spurious effects due to volume conduction may have excluded physiologically meaningful zero-lag synchrony (Finger et al., 2016). In addition, as MIM is based on imaginary coherence, it is insensitive to near-zero and 180° phase-lag interactions and may therefore discard physiologically meaningful coupling, while potentially emphasizing intermediate phase-lag components (e.g., around ±90°). Future work could test whether the observed effects replicate with alternative phase-based metrics that mitigate these biases, such as the debiased weighted phase-lag index (dwPLI; Vinck et al., 2011).

## 5 Conclusion

In summary, this study provides new insights into the effects of dopaminergic manipulation on EEG resting-state connectivity in healthy participants. Our findings show that, for phase ICMs, dopamine affects delta band and low-beta connectivity following a linear pattern, and that the gamma band follows a quadratic pattern. We also show that alpha and low-beta both have a linear effect on envelope ICMs. While our findings might be relevant for (the treatment of) psychotic symptoms, further studies should involve various antipsychotics and longitudinal designs.

## Data Availability

The raw data supporting the conclusions of this article will be made available by the authors, without undue reservation.

## Footnotes

1
https://www.uke.de/dateien/institute/neurophysiologie-und-pathophysiologie/downloads/meth_2019_04.zip

2
https://research.uni-leipzig.de/vigall/

3
https://www.r-project.org

4
https://posit.co/
