# Supplementary material for: Dopaminergic manipulation modulates frequency-specific EEG connectivity patterns: evidence from a single dose drug challenge study
Source: Front Neurosci. 2026 Mar 12;20:1734025. doi: 10.3389/fnins.2026.1734025 (PMC13017918; doi:10.3389/fnins.2026.1734025)
Supplement: Supplementary file 1 [file Data_Sheet_1.docx]

Supplementary Material


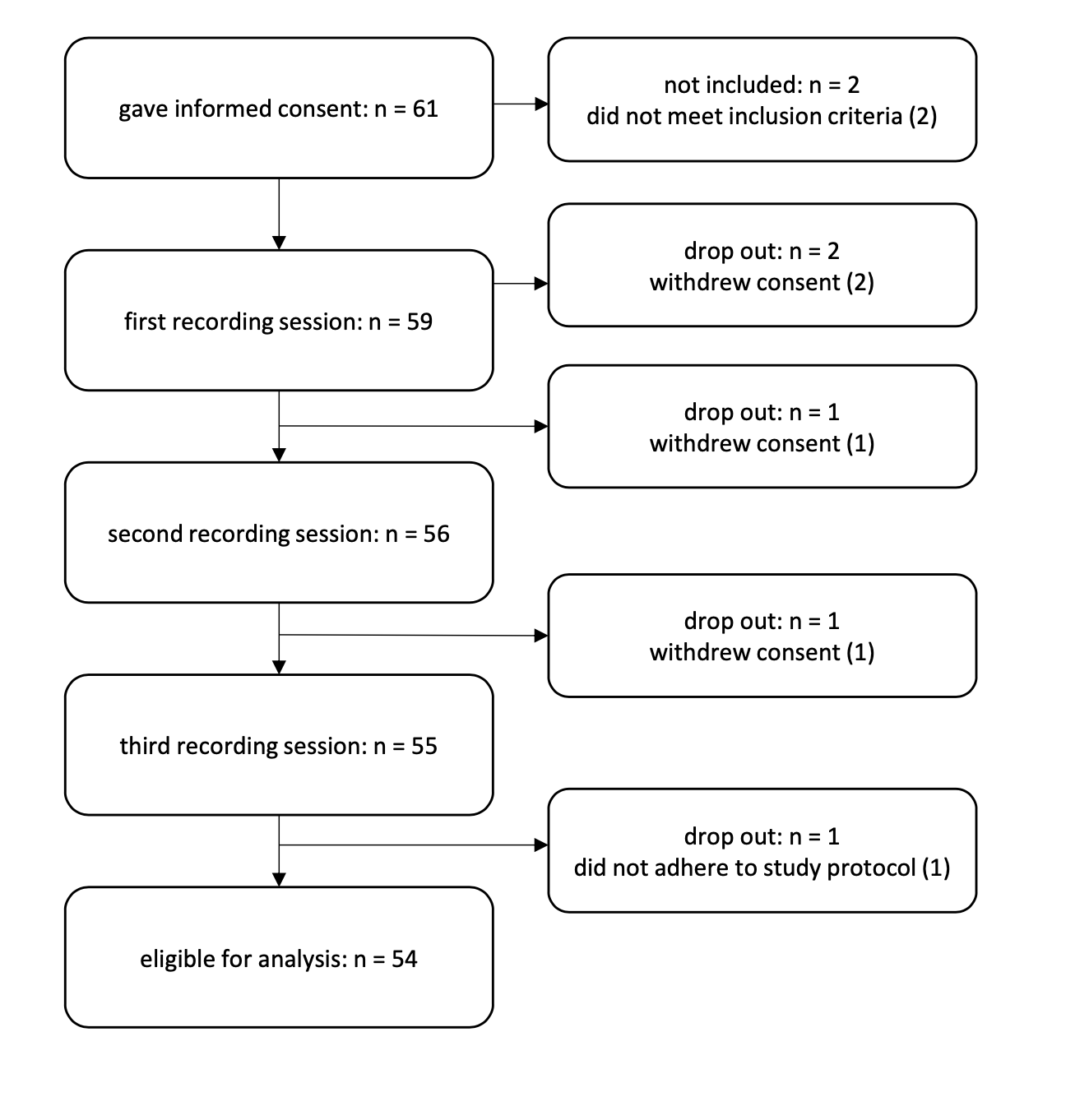


**Supplementary Figure 1.** Flowchart of participants eligible, recruited, numbers followed up and included in analysis.

**
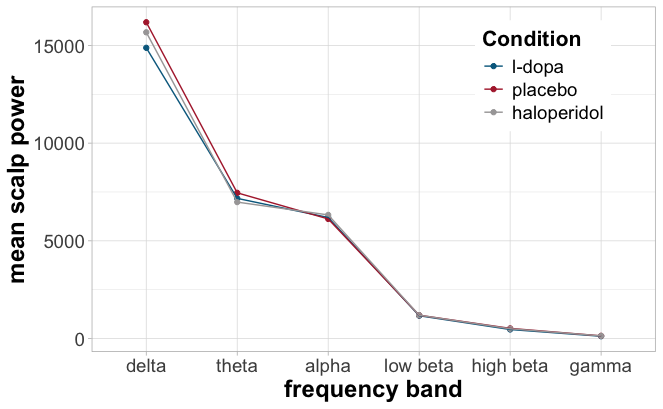
**

**Supplementary Figure 2.** Mean overall scalp power at each frequency band in L-dopa, haloperidol and placebo conditions.

| MIM | L-dopa | Placebo | Haloperidol |
| --- | --- | --- | --- |
| Delta (3 Hz) | 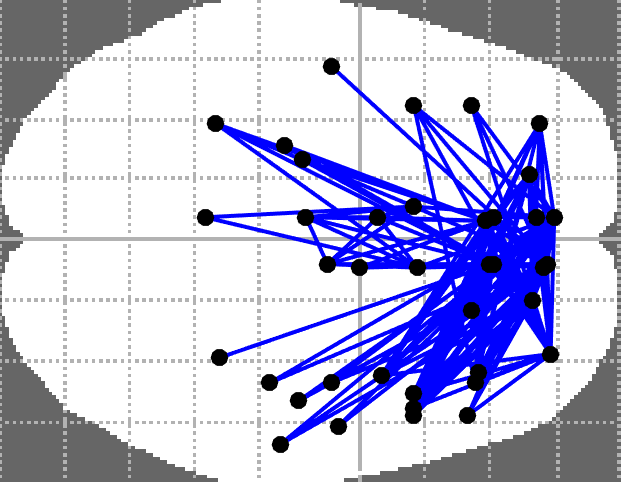 | 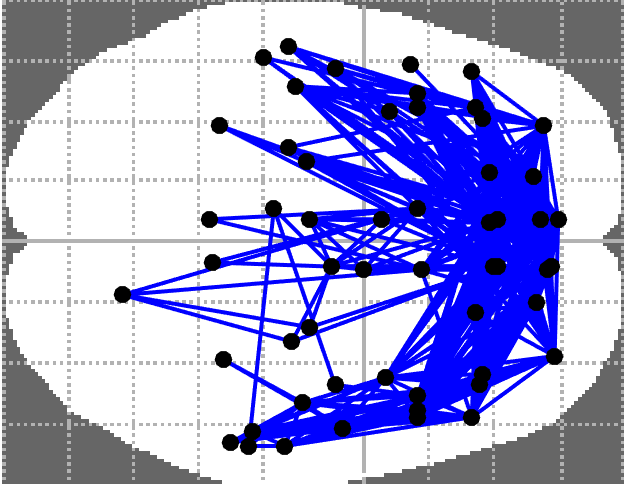 | 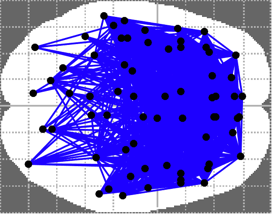 |
| Theta (6 Hz) | 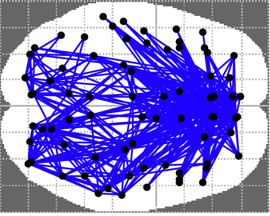 | 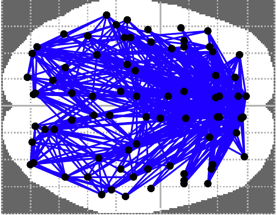 | 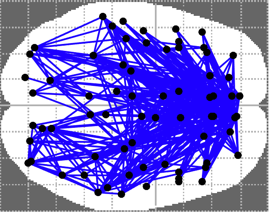 |
| Alpha (10 Hz) | 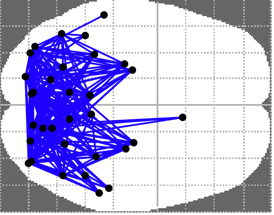 | 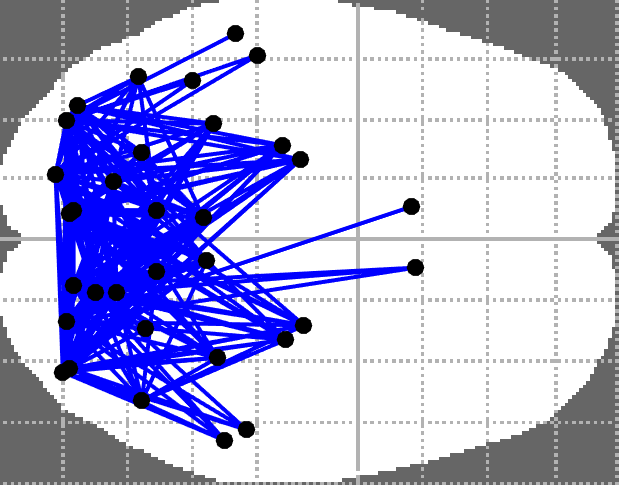 | 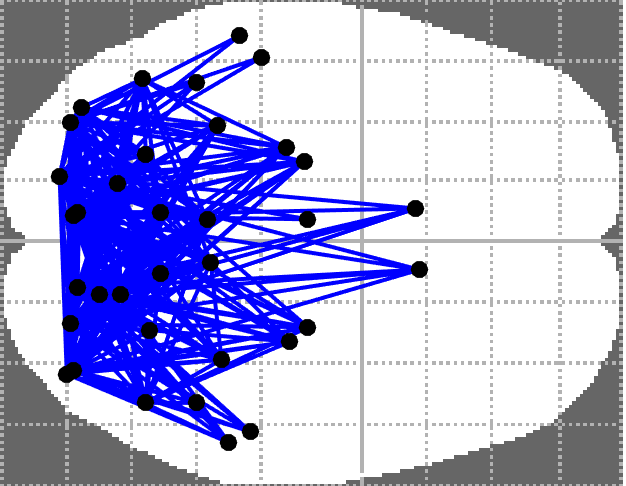 |
| Low beta (16 Hz) | 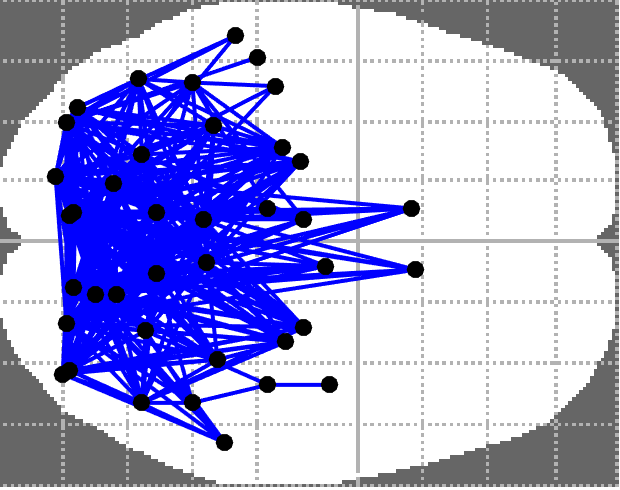 | 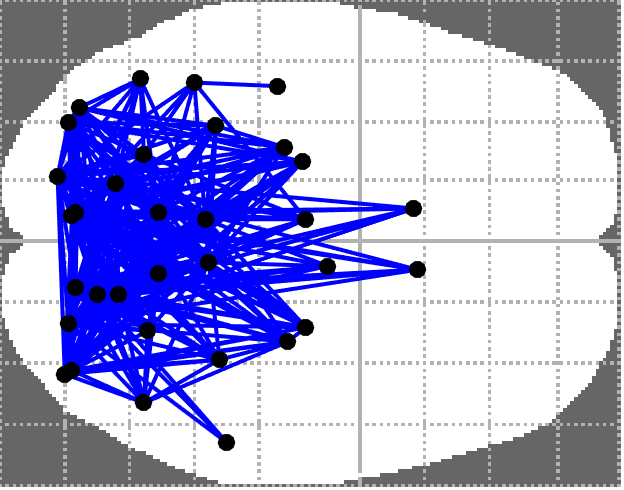 | 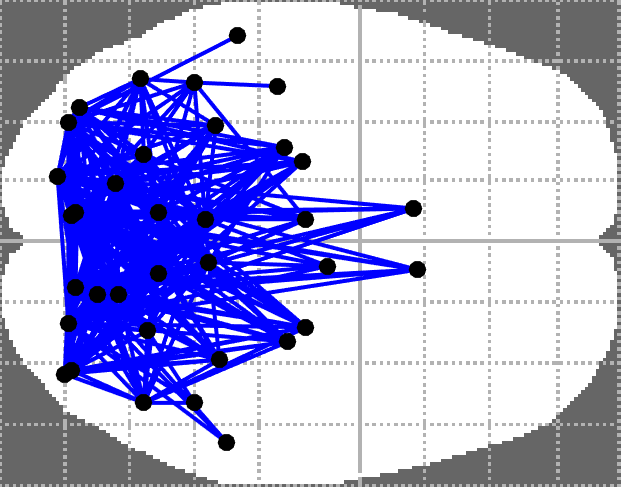 |
| High beta (25 Hz) | 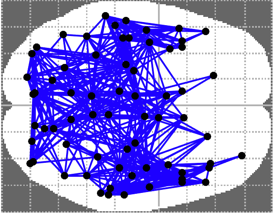 | 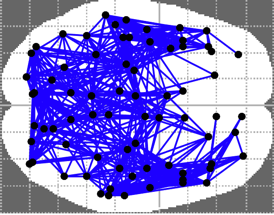 | 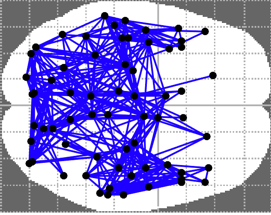 |
| Gamma (40 Hz) | 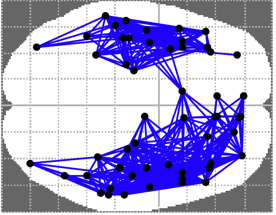 | 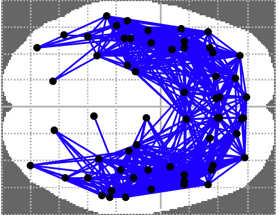 | 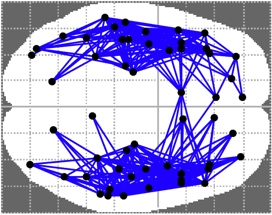 |

**Supplementary Figure 3**. Multivariate interaction measure (MIM) networks (one sample NBS t-test, 1000 permutations) in each frequency range. The t-threshold was varied across frequencies to maximize network visibility, but is the same across substances for any given frequency. The left side of the brain corresponds to the left hemisphere, and the top of the brain represents the anterior direction.

| ORT | L-dopa | Placebo | Haloperidol |
| --- | --- | --- | --- |
| Delta (3 Hz) | 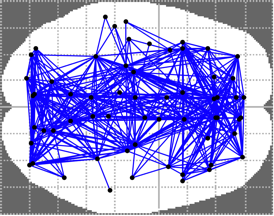 | 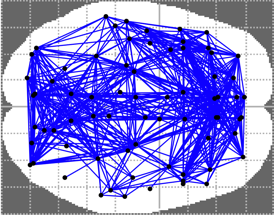 | 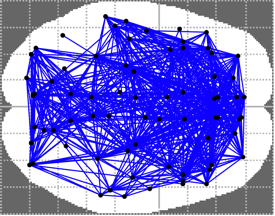 |
| Theta (6 Hz) | 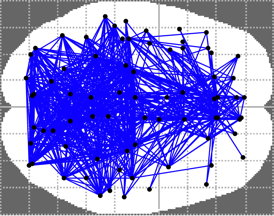 | 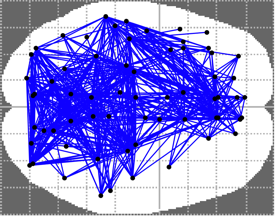 | 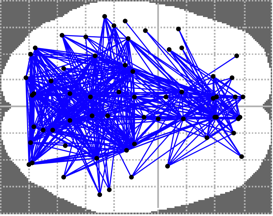 |
| Alpha (10 Hz) | 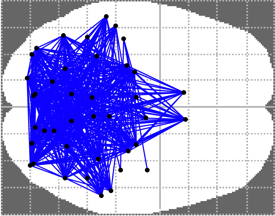 | 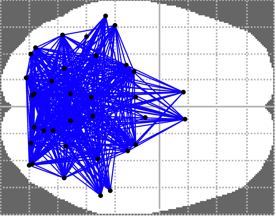 | 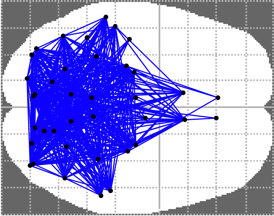 |
| Low beta (16 Hz) | 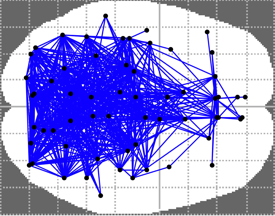 | 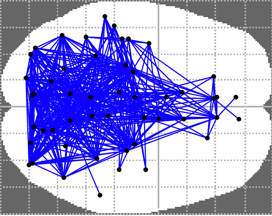 | 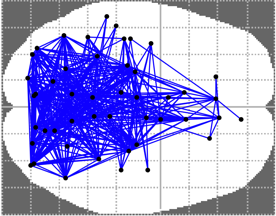 |
| High beta (25 Hz) | 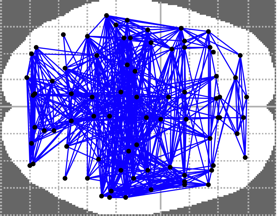 | 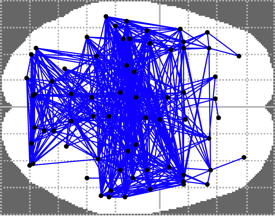 | 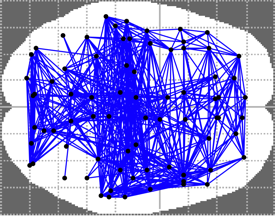 |
| Gamma (40 Hz) | 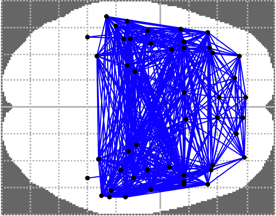 | 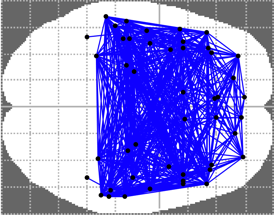 | 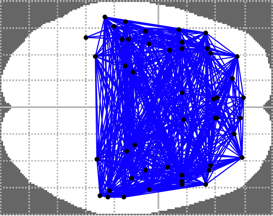 |

**Supplementary Figure 4**. Orthogonalized power envelope (ORT) networks (one sample NBS t-test, 1000 permutations) in each frequency range. The t-threshold was varied across frequencies to maximize network visibility, but is the same across substances for any given frequency. The left side of the brain corresponds to the left hemisphere, and the top of the brain represents the anterior direction.

**Supplementary Table 1**. Double-dummy design of the capsule administration

|  | |  | |  | |
| --- | --- | --- | --- | --- | --- |
|  | t0 | | t1 (1.5h after t0) | | t2 (2.5h after t0) |
| **Haloperidol** | Haldol® | | Placebo | | Onset EEG recording |
| **L-dopa** | Placebo | | Madopar® | | Onset EEG recording |
| **Placebo** | Placebo | | Placebo | | Onset EEG recording |

**Supplementary Table 2.** Brain regions included in significant networks with the number of connections within the network for delta, low-beta, and gamma for MIM.

**Delta**

| Node-Name (according to AALA) | Number of connections |
| --- | --- |
| Frontal_Inf_Tri_L | 1 |
| Frontal_Mid_L | 4 |
| Frontal_Mid_Orb_L | 1 |
| Frontal_Sup_L | 1 |
| Parietal_Inf_L | 2 |
| Postcentral_L | 3 |
| Precentral_L | 1 |
| SupraMarginal_R | 1 |

**Low-Beta**

| Node-Name (according to AALA) | Number of connections |
| --- | --- |
| Cingulum_Mid_L | 1 |
| Paracentral_Lobule_L | 1 |
| Paracentral_Lobule_R | 1 |
| Postcentral_L | 1 |
| Rolandic_Oper_R | 4 |

**Gamma**

| Node-Name (according to AALA) | Number of connections |
| --- | --- |
| Cingulum_Ant_L | 10 |
| Cingulum_Ant_R | 3 |
| Cingulum_Mid_R | 1 |
| Cingulum_Post_L | 2 |
| Frontal_Inf_Oper_L | 2 |
| Frontal_Inf_Orb_L | 4 |
| Frontal_Inf_Tri_L | 3 |
| Frontal_Med_Orb_L | 6 |
| Frontal_Med_Orb_R | 4 |
| Frontal_Mid_L | 8 |
| Frontal_Mid_Orb_L | 16 |
| Frontal_Sup_L | 3 |
| Frontal_Sup_Medial_L | 6 |
| Frontal_Sup_Medial_R | 6 |
| Frontal_Sup_Orb_L | 3 |
| Fusiform_L | 3 |
| Heschl_L | 3 |
| Hippocampus_L | 8 |
| Insula_L | 2 |
| Olfactory_L | 2 |
| Paracentral_Lobule_L | 2 |
| Paracentral_Lobule_R | 2 |
| Parahippocampal_L | 5 |
| Postcentral_L | 4 |
| Precentral_L is | 2 |
| Rectus_L | 4 |
| Rectus_R | 3 |
| Rolandic_Oper_L | 3 |
| Supp_Motor_Area_L | 1 |
| Supp_Motor_Area_R | 1 |
| Temp_Pole_Mid_L | 5 |
| Temp_Pole_Sup_L | 3 |
| Temporal_Sup_L | 1 |

**Supplementary Table 3.** Brain regions included in significant networks with the number of connections within the network for alpha and low-beta for orthogonalized power envelopes.

**Alpha**

| Node-Name (according to AALA) | Number of connections |
| --- | --- |
| Frontal_Inf_Oper_R | 2 |
| Frontal_Mid_R | 1 |
| Frontal_Sup_L | 1 |
| Postcentral_L | 3 |
| Precentral_L | 1 |
| Rolandic_Oper_R | 2 |

**Low-Beta**

| Node-Name (according to AALA) | Number of connections |
| --- | --- |
| Angular_L | 1 |
| Cingulum_Mid_L | 8 |
| Cingulum_Mid_R | 7 |
| Cingulum_Post_L | 3 |
| Cingulum_Post_R | 3 |
| Cuneus_L | 2 |
| Frontal_Inf_Oper_R | 15 |
| Frontal_Inf_Orb_L | 4 |
| Frontal_Mid_R | 11 |
| Frontal_Sup_L | 16 |
| Frontal_Sup_R | 3 |
| Frontal_Sup_Medial_R | 1 |
| Frontal_Sup_Orb_L | 1 |
| Fusiform_L | 1 |
| Heschl_L | 7 |
| Heschl_R | 11 |
| Hippocampus_L | 4 |
| Hippocampus_R | 1 |
| Insula_R | 13 |
| Lingual_L | 1 |
| Occipital_Mid_L | 2 |
| Occipital_Sup_L | 3 |
| Olfactory_L | 2 |
| Olfactory_R | 2 |
| Paracentral_Lobule_L | 6 |
| Paracentral_Lobule_R | 4 |
| Parahippocampal_L | 2 |
| Parahippocampal_R | 1 |
| Parietal_Inf_L | 6 |
| Parietal_Inf_R | 14 |
| Parietal_Sup_L | 6 |
| Parietal_Sup_R | 21 |
| Postcentral_L | 16 |
| Postcentral_R | 11 |
| Precentral_L | 6 |
| Precentral_R | 16 |
| Precuneus_L | 7 |
| Precuneus_R | 5 |
| Rolandic_Oper_L | 2 |
| Rolandic_Oper_R | 17 |
| Supp_Motor_Area_L | 11 |
| Supp_Motor_Area_R | 6 |
| SupraMarginal_L | 4 |
| SupraMarginal_R | 5 |
| Temporal_Sup_R | 2 |
